# Pharmacological Properties of Protocatechuic Acid and Its Potential Roles as Complementary Medicine

**DOI:** 10.1155/2015/593902

**Published:** 2015-02-09

**Authors:** Yoswaris Semaming, Patchareewan Pannengpetch, Siriporn C. Chattipakorn, Nipon Chattipakorn

**Affiliations:** ^1^Veterinary Technology Program, Faculty of Technology, Udon Thani Rajabhat University, Udon Thani 41000, Thailand; ^2^Cardiac Electrophysiology Research and Training Center, Faculty of Medicine, Chiang Mai University, Chiang Mai 50200, Thailand; ^3^Department of Pharmacology, Faculty of Medicine, Khon Kaen University, Khon Kaen 40002, Thailand; ^4^Department of Oral Biology and Diagnostic Science, Faculty of Dentistry, Chiang Mai University, Chiang Mai 50200, Thailand; ^5^Center of Excellence in Cardiac Electrophysiology Research, Chiang Mai University, Chiang Mai 50200, Thailand; ^6^Cardiac Electrophysiology Unit, Department of Physiology, Faculty of Medicine, Chiang Mai University, Chiang Mai 50200, Thailand

## Abstract

This paper reviews the reported pharmacological properties of protocatechuic acid (PCA, 3,4-dihydroxy benzoic acid), a type of phenolic acid found in many food plants such as olives and white grapes. PCA is a major metabolite of anthocyanin. The pharmacological actions of PCA have been shown to include strong *in vitro* and *in vivo* antioxidant activity. In *in vivo* experiments using rats and mice, PCA has been shown to exert anti-inflammatory as well as antihyperglycemic and antiapoptotic activities. Furthermore, PCA has been shown to inhibit chemical carcinogenesis and exert proapoptotic and antiproliferative effects in different cancerous tissues. Moreover, *in vitro* studies have shown PCA to have antimicrobial activities and also to exert synergistic interaction with some antibiotics against resistant pathogens. This review aims to comprehensively summarize the pharmacological properties of PCA reported to date with an emphasis on its biological properties and mechanisms of action which could be therapeutically useful in a clinical setting.

## 1. Introduction

Protocatechuic acid (PCA, 3,4-dihydroxybenzoic acid) is a phenolic compound found in many food plants such as* Olea europaea* (olives),* Hibiscus sabdariffa* (roselle),* Eucommia ulmoides* (du-zhong),* Citrus microcarpa* Bunge (calamondin), and* Vitis vinifera* (white wine grapes) [1–3]. PCA content varies considerably depending on the type of food.

Recently, several investigations have shown that PCA is a major metabolite of complex polyphenols, especially anthocyanins [4, 5]. Anthocyanins have been shown to affect a variety of physiological activities which are of great benefit to health, including a reduced risk of cardiovascular diseases. This particular beneficial effect is partly due to the anti-inflammatory properties [6–8], antioxidant and free radical scavenging activities [9–12], peroxidation inhibition [13], and estrogenic/antiestrogenic activity [14] of PCA. PCA is of particular nutritional interest since it is a main anthocyanin metabolite that can reach tissues in amounts which can exert biological effects on health [15].* In vivo* studies demonstrated that male balb/cA mice which were fed a standard diet supplemented with PCA for 12 weeks showed increased PCA levels in plasma and tissues such as brain, heart, liver, and kidney [16]. Moreover, PCA itself has been shown to possess antioxidant properties as well as having other potential health benefits such as anti-inflammatory effects.

The aim of this review is to comprehensively summarize the pharmacological properties of PCA reported to date including antioxidant, anti-inflammatory, antihyperglycemia, antiapoptosis/proapoptosis, and antimicrobial activities, with an emphasis on the biological properties and mechanisms of action which could be potentially useful in a clinical setting.

## 2. Antioxidant Activity of PCA

Oxidative stress plays a key role in the pathogenesis of degenerative diseases such as cardiovascular diseases, diabetes mellitus, neurodegenerative diseases, cancer, and aging [17–21]. Mounting evidence from both* in vitro* and* in vivo* studies demonstrates that PCA exerts potent antioxidative effects. In* in vitro* studies, as summarized in Table 1, PCA was shown to have free radical scavenging and antioxidant activities by decreasing lipid peroxidation and increasing the scavenging of hydrogen peroxide (H_2_O_2_) and diphenylpicrylhydrazyl (DPPH) [22]. In J77A.1 macrophage, PCA decreased oxidized low-density lipoprotein levels (LDL), inhibited superoxide (O_2_^•^) and H_2_O_2_ production, and also restored glutathione (GSH) related enzymes via c-Jun N-terminal kinase (JNK) mediated nuclear factor (erythroid-derived 2) like 2 (Nrf2) activation [23, 24]. PCA also reduced reactive oxygen species (ROS) induced apoptosis by improving mitochondrial function, inhibiting DNA fragmentation in H_2_O_2_-induced oxidative stress in human neuronal cells [25], preventing lactate dehydrogenase (LDH) release in H_2_O_2_-induced oxidative stress in PC12 cells [26], and inhibiting intracellular ROS level in BNLCL2 cells [27].

Consistent with* in vitro* reports,* in vivo* studies (as summarized in Table 2) also demonstrated that PCA treatment decreased oxidative stress by promoting endogenous antioxidant enzymes in aging rats and also reduced H_2_O_2_-induced oxidative damage in aging mice, thus indicating that PCA could prevent oxidative damage in aging animals [26, 28]. PCA also decreased advance glycation end products (AGEs) and ROS production in D-galactose-induced ROS and AGEs formation in mice [29]. In streptozotocin (STZ) induced diabetic rats, PCA was also found to decrease ROS formation in liver, heart, kidney, and brain by restoring endogenous antioxidant enzyme activities [3, 30]. All of these findings indicated that the PCA possess potential antioxidant activity, suggesting that it could be used as a complementary medication to prevent oxidative damage in various degenerative diseases.

## 3. Anti-Inflammatory Activity of PCA

The inflammatory process is regulated by coordinated activation of both pro- and anti-inflammatory mediators in tissue cells (such as fibroblasts, endothelial cells, tissue macrophages, and mast cells) and also by the recruitment of leucocytes [31, 32]. Prolonged activation of proinflammatory mediators causes tissue injury and organ dysfunction. As a consequence, chronic inflammation plays a critical role in the pathophysiology of major chronic diseases including obesity, cardiovascular disease, diabetes mellitus, Alzheimer's disease, and many types of cancer [33, 34]. The mediators, including nitric oxide (NO), lipid mediators, cytokines/chemokines, adhesion molecules, and matrix metalloproteinases (MMPs), are involved in the initiation, maintenance, and resolution of the inflammatory process [35, 36].

A summary of* in vitro* studies regarding the effects of PCA on the inflammatory process is shown in Table 3. PCA was shown to suppress tumor necrosis factor alpha (TNF-*α*), interleukin- (IL-) 1*β*, inducible nitric oxide synthase (iNOS), and cyclooxygenase 2 (COX-2) expression via the regulation of the nuclear factor kappa-light-chain-enhancer of activated B cells (NF-kB) and mitogen-activated protein kinase (MAPK) activation in lipopolysaccharide- (LPS-) induced RAW 264.7 cell damage [37]. Moreover, PCA also suppressed vascular cell adhesion protein 1 (VCAM-1) and intercellular adhesion molecule 1 (ICAM-1) mRNA expression in TNF-*α*-induced cellular damage [38] and inhibited monocyte infiltration [39].

Consistent with* in vitro* reports, animal studies (Table 4) demonstrated that PCA strongly inhibited inflammation by inhibiting carrageenan-induced inflammation in mice by decreasing TNF-*α*, IL-1*β*, and prostaglandin E2 (PGE_2_) levels, suppressed iNOS and COX-2 expression in apolipoprotein E (ApoE) deficient mice [37], prevented LPS-induced sepsis in mice via decreased NO levels and suppressed IL-10 [40], reduced VCAM-1 and ICAM-1 [38], and inhibited monocyte/macrophage infiltration in mice [39]. Moreover, PCA also prevented coagulation and inflammation in STZ-induced diabetic rats by inhibiting the plasma levels of the plasminogen activator inhibitor 1 (PAI-1), antithrombin III (AT-III), protein C, C-reactive protein (CRP), and von Willebrand factor (vWF) and reduced IL-6, TNF-*α*, and monocyte chemoattractant protein-1 (MCP-1) levels in the heart and kidneys [3]. These findings suggest that the anti-inflammatory effects of PCA might be beneficial in various chronic degenerative diseases in which the inflammatory process plays an important part in the pathogenesis.

## 4. Antihyperglycemic Activity of PCA

Maintenance of glucose homeostasis by strict hormonal control is of the utmost importance to human physiology [41, 42]. Failure of the control of glucose levels, with defects in both insulin action and insulin secretion, can result in a metabolic syndrome which is a multisymptom disorder of energy homeostasis [43]. It has been demonstrated that peroxisome proliferator-activated receptor gamma (PPAR*γ*) is one of several targets of insulin activity, which regulates the expression and activity of key players in the maintenance of glucose transport machinery efficiency, such as glucose transporter (GLUT) 4 and adiponectin [44, 45]. In* in vitro* studies, as summarized in Table 5, PCA has been shown to exert an insulin-like activity in oxidized LDL-induced insulin resistance in adipocytes via increased PPAR*γ* activation [45]. Similarly,* in vivo* studies (Table 6) also demonstrated that PCA decreased blood glucose levels in STZ-induced diabetes via restored carbohydrate metabolic enzyme activity, increased plasma insulin level, and normalized the activity of pancreatic islets [3, 30, 46]. These findings suggest that PCA provides antihyperglycemic effects in addition to its reported antioxidant and anti-inflammatory effects.

## 5. Antiapoptosis versus Proapoptotic Activity of PCA

Polyphenols have been shown to improve cell survival and protect against cytotoxicity by inhibiting apoptosis [18]. However, they can also induce apoptosis and prevent tumor growth [47, 48]. These opposite effects are mainly due to its effects on the controlling of the cell redox state. Evidence from* in vitro* studies (Table 7) revealed that PCA has cell-protective effects via increased IkB degradation and subsequent NF-kB activation in TNF-*α*-induced cell death [49], attenuated changes of the mitochondrial membrane permeability, decreased oxidative stress damage and increased Bcl-2 levels in 1-methyl-4-phenylpyridinium- (MPP^+^-) induced apoptotic cell death [50], decreased caspase-3 activity in isolated neuronal stem cells (NSCs) [51], and reduced LDH leakage in H_2_O_2_-induced apoptosis [52]. In MPP^+^-induced cell death, PCA treatment resulted in a return to normal cellular morphology and normal mitochondria [53]. Moreover, PCA has been shown to have cell-protective effects via antioxidant and scavenging activities [54].

Unlike the cells described in Table 7, evidence from cancer cell studies (Table 8) demonstrated that PCA can induce apoptosis and prevent the growth of tumor cells via causing reduced Bcl-2 protein, increased Bax protein expression in human leukemia (HL-60) cells [55], via activated JNK/p38 MAPK pathways and Fas/FasL pathways, increased translocation of Bax, and reduced Bcl-2 levels in human gastric adenocarcinoma cells [56] and via induced JNK and p38 MAPK pathways in HepG2 hepatocellular carcinoma cells [57]. Moreover, PCA also demonstrated anticancer properties by causing apoptosis or suppressing invasion and metastasis in human breast, lung, liver, cervix and prostate cancer cells [58]. Consistently, an* in vivo* study (Table 9) also demonstrated that PCA inhibited* N*-nitrosomethylbenzylamine (NMBA) induced esophageal tumorigenesis by its inhibitory effects on genes associated with inflammation in rats [59].

## 6. Antimicrobial Activity of PCA

*In vitro* studies (Table 10) demonstrated that PCA has an antimicrobial effect against gram positive and negative bacteria and fungi [60, 61]. PCA also prevented contamination of meat by* Campylobacter* and aerobes, by decreasing lipid oxidation [62]. PCA exerted its antibacterial effects due to its ability to inhibit bacterial growth and increase the synergistic effects of antibiotics hence reducing the possibility of resistance to drugs [63]. These antimicrobial activities of PCA have been proposed as promising applications in both health protection and food preservation in order to avoid food-borne illnesses [62, 64].

## 7. Conclusion

Growing evidence suggests the significant biological potential of PCA through the modulation of cellular signals involved in the control of oxidative stress and inflammation. Moreover, its antiapoptotic effects in normal cells and proapoptotic effects in cancer cells suggest definite benefits as a potential chemotherapeutic agent. However, much evidence of such properties has been collected from cellular and animal studies, while clinical studies are still lacking. Future clinical studies are needed to warrant the clinical usefulness of the PCA.

## Figures and Tables

**Table 1:** Summary of *in vitro* studies of antioxidant activities of PCA.

Model	Method	PCA concentration	Major finding	Interpretation	Reference
Biochemical assay	(i) TBAR assay (ii) H_2_O_2_ assay (iii) DPPH^.^ assay	0.05 and 0.10 mg/mL	(i) PCA increased % inhibition of lipid peroxidation (ii) PCA increased % scavenging of H_2_O_2_ (iii) PCA increased % scavenging of DPPH^.^	PCA exerted antioxidant activity	[22]

J774 A.1 macrophages	J774 A.1 macrophages	3 and 25 mol/L	(i) PCA decreased oxidation of LDL (ii) PCA inhibited O_2_ and H_2_O_2_ production (iii) PCA increased GSH content (iv) PCA restored GR and GPx activities (v) PCA restored the *γ*-GCS mRNA, GR, and GPx expression	PCA had an antioxidant activity via activation of mRNA transcription of GSH-related enzymes	[23]

J774 A.1 macrophages	Direct PCA application to cells	25 *μ*M	(i) PCA increased GSH, GPx, and GR expression (ii) PCA increased Nrf2 expression and activation (iii) PCA increased JNK mRNA level	PCA increased macrophage endogenous antioxidants via JNK-mediated Nrf2 activation	[24]

Human neuronal cell line	H_2_O_2_-induced oxidative stress	25, 50, and 100 *μ*M	(i) PCA inhibited ROS formation at cytosolic level (ii) PCA inhibited apoptotic events (iii) PCA improved mitochondrial function (iv) PCA decreased DNA fragmentation	PCA reduced apoptosis via ROS reduction, improved mitochondrial function, and inhibited DNA fragmentation	[25]

PC12 cells	H_2_O_2_-induced oxidative damage	50, 100, 150, and 200 *μ*M	(i) PCA increased cell viability (ii) PCA decreased % LDH release	PCA prevented H_2_O_2_-induced cell death	[26]

BNLCL2 cells	H_2_O_2_-induced oxidative damage	1, 5, 10, 20, and 100 *μ*g/mL	(i) PCA affected DPPH scavenging activity (ii) PCA inhibited liposome peroxidation (iii) PCA reduced intracellular ROS level	PCA had a radical scavenging activity and antioxidant property	[27]

**Table 2:** Summary of *in vivo* studies of antioxidant activities of PCA.

Model	Method	PCA dose/route/duration	Major finding	Interpretation	Reference
Sprague-Dawley rat	STZ-induced T1DM (50 mg/kg, ip)	50, 100 mg/kg, po	(i) PCA decreased plasma MDA (ii) PCA decreased cardiac MDA (iii) PCA decreased mitochondrial ROS production	PCA deceased oxidative stress in T1DM rats	[30]

Sprague-Dawley rat	H_2_O_2_-induced oxidative damage in young and age rats	5 mg/kg/day for 7 days (ip)	(i) PCA improved scores during the passive avoidance testing (ii) PCA decreased MDA in brain of aged rat (iii) PCA increased GSH-PX activity	PCA promoted endogenous antioxidant enzymatic activities and inhibited ROS generation	[26]

Mice	D-galactose-induced ROS and AGEs	0.5%, 1%, or 2% in diet for 8 weeks	(i) PCA decreased ROS and protein carbonyl content (ii) PCA retained GSH content (iii) PCA decreased CML, pentosidine, sorbitol, fructose, and methylglycoxal level in brain	PCA had antiglycative and antioxidant activity by retaining GSH	[29]

Mice	Young and aged	5 and 10 mg/kg (ip) for 7 days	In aged rats (i) PCA elevated splenic weight (ii) PCA increased the activities of GSH-PX (iii) PCA increased catalase (CAT) activity (iv) PCA decreased malondialdehyde (MDA) level	PCA was a potential antiageing agent by promoting endogenous antioxidant enzymatic activities	[28]

Mice	STZ-induced DM (50 mg/kg/iv)	1%, 2%, and 4% in diet for 8 weeks	(i) PCA at all concentrations decreased cardiac and renal MDA level (ii) PCA at 2% and 4% increased cardiac and renal GSH level (iii) PCA at 2% and 4% decreased cardiac and renal GSSG formation (iv) PCA at 2% and 4% increased GPX and catalase activity in cardiac and renal tissues	PCA had an antioxidative effect through the restoration of endogenous antioxidants	[3]

**Table 3:** Summary of *in vitro* studies of anti-inflammatory activities of PCA.

Model	Method	PCA concentration	Major finding	Interpretation	Reference
RAW 264.7 cells	Lipopolysaccharide- (LPS-) induced cellular damage	1, 2, 5, and 25 *μ*M	(i) PCA decreased TNF-*α* and IL-1*β* (ii) PCA decreased NO and PGE_2_ (iii) PCA inhibited iNOS and COX-2 expression (iv) PCA inhibited IkB-*α* degradation (v) PCA inhibited NF-kB phosphorylation (vi) PCA inhibited p38, ERK, and JNK	PCA had anti-inflammatory effects by regulating NF-kB and MAPK activation	[37]

Mouse aortic endothelial cell (MAEC)	TNF-*α*-induced cellular damage	0.05, 0.5, 5.0, 10, 20, and 40 *μ*mol/L	(i) PCA inhibited adhesion of HL-60 cells to MAECs (ii) PCA suppressed VCAM-1 and ICAM-1 mRNA expression (iii) PCA reduced NF-kB activation	PCA had an anti-inflammatory effect by inhibiting monocyte adhesion molecules	[38]

Cell culture	Isolated peripheral blood monocytes (PBMs) from ApoE-deficient mice	0.125, 0.25, and 0.5 *μ*mol/L	(i) PCA decreased CCR2 protein and mRNA expression (ii) PCA inhibited mouse PBMs migration	PCA exerted antiatherogenic properties by inhibiting monocyte infiltration	[39]

**Table 4:** Summary of *in vivo* studies of anti-inflammatory activities of PCA.

Model	Method	PCA dose/route/duration	Major finding	Interpretation	Reference
Mice	Carrageenan-induced inflammation in BALB/c mice	5 and 25 mg/kg, po (24 h)	(i) PCA reduced exudate (ii) PCA decreased protein content (iii) PCA reduced leukocyte number (iv) PCA inhibited TNF-*α*, IL-1*β*, and PGE_2_ level (v) PCA inhibited COX-2 and NF-kB expression	PCA exerted anti-inflammatory effects by inhibiting NF-kB activation.	[37]

Mice	ApoE-deficient mice	0.033% (w/w) of diet for 20 weeks	(i) PCA reduced sinus plague area (ii) PCA decreased cholesterol accumulation in aortas (iii) PCA reduced VCAM-1 and ICAM-1 expression in aortas (iv) PCA reduced NF-kB binding activity (v) PCA reduced plasma-soluble VCAM-1 and ICAM-1 levels	PCA exerted antiatherosclerosis effects by inhibiting adhesion molecules and reducing NF-kB activation	[38]

Mice	Thioglycollate-induced peritonitis in ApoE-deficient mice	25 mg/kg (po) for 11 days	(i) PCA decreased CCR2 protein and mRNA expression in PBMs of ApoE-deficient mice (ii) PCA reduced macrophage infiltration into the abdominal cavity	PCA exerted antiatherogenic properties by inhibiting monocyte/macrophage infiltration	[39]

Mice	LPS-induced sepsis (20 mg/kg, ip)	50 mg/kg (ip) single dose	(i) PCA reduced lethality (ii) PCA suppressed TNF-*α* and IL-10 (iii) PCA decreased plasma ALT levels (iv) PCA decreased plasma nitrite/nitrate levels (v) PCA decreased hepatic MDA levels	PCA exerted sepsis prevention properties by inhibiting inflammatory cytokines and antioxidant activity	[40]

Mice	STZ-induced DM (50 mg/kg/iv)	1%, 2%, and 4% in diet for 8 weeks	(i) PCA lowered plasma PAI-1 levels (ii) PCA increased plasma AP-III levels (iii) PCA increased plasma protein C levels (iv) PCA lowered plasma CRP levels (v) PCA decreased plasma von Willebrand factor (vi) PCA reduced IL-6, TNF-*α*, and MCP-1 levels in heart and kidney	PCA exerted anticoagulatory and anti-inflammatory effects by lowering inflammatory cytokines	[3]

**Table 5:** Summary of *in vitro* study of antihyperglycemic activities of PCA.

Model	Model/method	PCA concentration	Major finding	Interpretation	Reference
Human omental adipocytes and murine adipocyte 3T3-L1 cells	oxLDL-induced insulin resistance	100 *μ*M	(i) PCA increased glucose uptake (ii) PCA increased GLUT4 translocation (iii) PCA increased PPAR*γ* activity (iv) PCA increased adiponectin	PCA exerted an insulin-like activity in adipocytes by increasing PPAR*γ* activation	[45]

**Table 6:** Summary of *in vivo* studies of antihyperglycemic activities of PCA.

Model	Model/method	PCA dose/route/duration	Major finding	Interpretation	Reference
Sprague-Dawley rat	STZ-induced T1DM (50 mg/kg, ip)	50, 100 mg/kg (po)	(i) PCA decreased FBG (ii) PCA decreased HbA_1c_	PCA exerted hypoglycemic effects in T1DM	[30]

Mice	STZ-induced DM (50 mg/kg, iv)	1%, 2%, and 4% in diet for 8 weeks	(i) PCA lowered plasma glucose levels (ii) PCA increased insulin levels (iii) PCA decreased TG and TC content in plasma, heart, and liver	PCA attenuated diabetic conditions by lowering plasma glucose, increasing insulin, and lowering triglyceride levels	[3]

Sprague-Dawley rat	STZ-induced DM (40 mg/kg, ip)	50, 100, 200 mg/ kg/day (po) for 45 days	(i) PCA decreased plasma glucose levels (ii) PCA decreased HbA_1c_ levels (iii) PCA increased plasma insulin levels (iv) PCA increased hexokinase activity and increased glycogen content in liver (v) PCA decreased activity of glucose 6-phosphatase and fructose 1,6-bisphosphatase in liver (vi) PCA reduced adipose tissue of DM pancreas and normalized pancreatic islets	PCA exerted antihyperglycemic effects by restoring carbohydrate metabolic enzyme activity and increasing plasma insulin levels	[46]

Mice	STZ-induced DM (50 mg/kg, iv)	2% and 4% in diet for 12 weeks	(i) Content of PCA increased in plasma, brain, heart, liver, and kidney (ii) PCA decreased water intake and food intake (iii) PCA increased body weight (iv) PCA decreased urine volume (v) PCA reduced plasma glucose levels (vi) PCA increased plasma insulin levels (vii) PCA decreased plasma BUN level (viii) PCA increased creatinine clearance rate (ix) PCA decreased HbA_1C_ level (x) PCA decreased urine glycated albumin (xi) PCA reduced renal production of CML, pentosidine, sorbitol, and fructose (xii) PCA decreased brain content of CML, pentosidine, fructose, and sorbitol (xiii) PCA decreased urinary albumin (xiv) PCA reduced level of fibronectin, type-IV collagen, and TGF-*β* in renal tissue (xv) PCA reduced renal activity and expression of AR and SDH (xvi) PCA increased renal activity and expression of GLI (xvii) PCA decreased renal activity and mRNA expression of PKC-*α* and PKC-*β* (xviii) PCA decreased RAGE mRNA expression	PCA had an antihyperglycemic, antiglycative and renoprotective effects via increasing plasma insulin, reducing plasma glucose, reducing renal level of glycation end products, fibronectin, TGF-*β*, and repressing renal activity and expression of AR, SDH, GLI, PKC-*α*, PPAR-*γ*, restoring PPAR-*γ*, and suppressing RAGE	[16]

**Table 7:** Summary of *in vitro* studies of antiapoptotic activities of PCA in noncancer cells.

Model	Model/method	PCA concentration	Major finding	Interpretation	Reference
HUVECs and Jurkat cells	TNF-*α*-induced cell death	50, 100 *µ*M and 1 nM	(i) PCA inhibited TNF-*α*-induced HUVECs and Jurkat cells death (ii) PCA increased NF-kB activation (iii) PCA increased degradation of IkB	PCA exerted cell-protective effects via increased IkB degradation and subsequent NF-kB activation	[49]

PC12 cells	MPP^+^-induced mitochondrial dysfunction and apoptotic cell death	0.3, 0.6, and 1.2 mM	(i) PCA reduced the number of cell death (ii) PCA at 0.6 and 1.2 mM decreased percentage of depolarized cell, reduced ROS formation, and increased GSH content (iii) PCA at 0.6 and 1.2 mM decreased caspase-3 activity and increased Bcl-2 protein	PCA exerted antiapoptotic activities via attenuated changes of mitochondrial membrane permeability and decreased oxidative stress damage	[50]

Isolated NSCs of embryonic rat	Direct PCA application to cells	0.006, 0.03, 0.06, and 0.12 mM	(i) PCA at 0.03, 0.06, and 0.12 mM increased cellular viability (ii) PCA reduced nuclear fragmentation (iii) PCA reduced the levels of apoptosis (iv) PCA decreased endogenous ROS level (v) PCA decreased caspase-3 activity	PCA inhibited cell apoptosis via suppression of the caspase cascade	[51]

PC12 cells	H_2_O_2_-induced apoptosis	0.006, 0.03, 0.06, and 0.12 mM	(i) PCA (over 0.3 mM) increased cellular viability (ii) PCA reduced LDH leakage (iii) PCA reduced apoptotic sub-G1 population	PCA promoted cell viability and inhibited apoptotic cell death	[52]

PC12 cells	MPP^+^-induced apoptotic cell death	0.33, 0.65, and 1.30 mM	(i) PCA reduced cell death in a dose-dependent manner (ii) PCA treatment exhibited normal cellular morphology and normal mitochondria (iii) PCA increased tyrosine hydroxylase expression (iv) PCA reduced oligomeric *α*-synuclein (v) PCA increased monomeric *α*-synuclein	PCA had neuroprotective effects via reducing cell death and inhibiting oligomerization of *α*-synuclein	[53]

Rat primary hepatocytes	t-BHP (1.5 mM) induced oxidative damage	0.02, 0.05, and 0.10 mg/mL	(i) PCA 0.05 and 0.10 mg/mL decreased LDH, ALT, and MDA (ii) PCA prevented mitochondrial depolarization (iii) PCA increased scavenging activity on DPPH	PCA had a cell-protective effect via its antioxidant and scavenging activity	[54]

**Table 8:** Summary of *in vitro* studies of proapoptotic activity of PCA in cancer cells.

Model	Model/method	PCA concentration	Major finding	Interpretation	Reference
HL-60 leukemia cells	Direct PCA application to cells	0.2, 0.5, 1, and 2 mM	(i) PCA increased DNA fragmentation (ii) PCA declined hyperphosphorylated RB level (iii) PCA reduced Bcl-2 protein expression (iv) PCA increased Bax protein expression	PCA had an antiproliferative effect via induced RB phosphorylation and degradation and Bcl-2 protein suppression in cancer cells	[55]

Human gastric adenocarcinoma (AGS) cells	Direct PCA application to cells	0.5, 1.0, 2.0, 4.0, 6.0, 8.0, and 9.0 mM	(i) PCA increased apoptotic bodies formation (ii) PCA increased p-JNK expression (iii) PCA increased p-p53 expression (iv) PCA increased phosphorylation of ATF-2 at Thr^69/71^ and c-Jun at Ser^73^ (v) PCA increased Fas expression (vi) PCA increased FasL expression (vii) PCA decreased Bcl-2 expression (viii) PCA increased Bax expression	PCA induced apoptosis via JNK/p38 MAPK pathway, activated Fas/FasL pathway, increased translocation of Bax, and reduced Bcl-2 in cancer cells	[56]

HepG2 hepatocellular carcinoma cells	Direct PCA application to cells	0, 3, 10, 30, 100, and 300 *μ*mol/L	(i) PCA decreased viability of HepG2 hepatocellular carcinoma (ii) PCA increased JNK and p53 expression	PCA induced cell death via activating JNK and p38 MAPK pathways in cancer cells	[57]

Human breast, lung, liver, cervix, and prostate cancer cells	Direct PCA application to cells	1, 2, 4, and 8 *μ*mol/L	(i) PCA decreased viability (ii) PCA enhanced DNA fragmentation (iii) PCA decreased MMP (iv) PCA lowered Na^+^-K^+^-ATPase activity (v) PCA increased caspase-3 activity (vi) PCA increased caspase-8 activity (vii) PCA decreased ICAM-1 level (viii) PCA at 2, 4, and 8 *μ*mol/L decreased VEGF level (ix) PCA suppressed IL-6 and IL-8 levels	PCA had anticancer properties via increased apoptosis or suppressed invasion and metastasis cancer cells	[58]

**Table 9:** Summary of *in vivo* studies of proapoptotic activity of PCA.

Model	Method	PCA dose/route/duration	Major finding	Interpretation	Reference
Rat	NMBA-induced esophageal cancer in rats	PCA 0.05% in diet for 15, 25, and 35 weeks	(i) PCA reduced area of hyperplasia began at week 25 (ii) PCA reduced COX-2, iNOS, soluble epoxide hydrolase (she), and pentraxin-3 (PTX3) mRNA expression levels	PCA prevented esophageal tumorigenesis, by inhibitory effects on genes associated with inflammation	[59]

**Table 10:** Summary of *in vitro* studies of antimicrobial activity of PCA.

Model	Method	PCA concentration	Major finding	Interpretation	Reference
*Campylobacter spp *	Antimicrobial activity testing	(i) 10 mg/mL (ii) 5, 10 mg/100 g beef	(i) PCA inhibited growth and susceptible and antibiotic-resistant *Campylobacter species* (ii) PCA inhibited growth of aerobes in beef samples (iii) PCA decreased lipid oxidation levels in ground beef	(i) PCA could preserve foods to prevent contamination by *Campylobacter* and aerobes, via decreased lipid oxidation	[62]

*Pseudomonas aeruginosa *	Antimicrobial susceptibility testing	2,000 *μ*g/mL	(i) PCA inhibited growth of *Pseudomonas aeruginosa* (ii) PCA plus sulfamethoxazole increased synergistic mode of inhibition of *P*. *aeruginosa *	(i) PCA had an antibacterial effect by inhibiting bacterial growth and increasing the synergistic effects on antibiotics to reduced drug resistance	[63]

Bacteria and fungi	Antimicrobial activity testing	1.22–625 *μ*g/mL	(i) PCA prevented 80% of the growth of organisms	(i) PCA had an antimicrobial effect against gram positive and negative bacteria and against fungi	[60]

*Helicobacter pylori *	Antimicrobial susceptibility testing	8–64 mg/L	(i) PCA inhibited growth of *H*. *pylori* (ii) PCA reduced drug-resistant *H*. *pylori* (iii) PCA at (32–40 mg/L) reduced urease activity of *H*. *pylori* to 40%	(i) PCA had growth prevention effects on *H*. *pylori *	[61]
